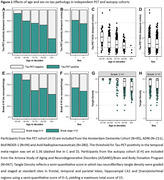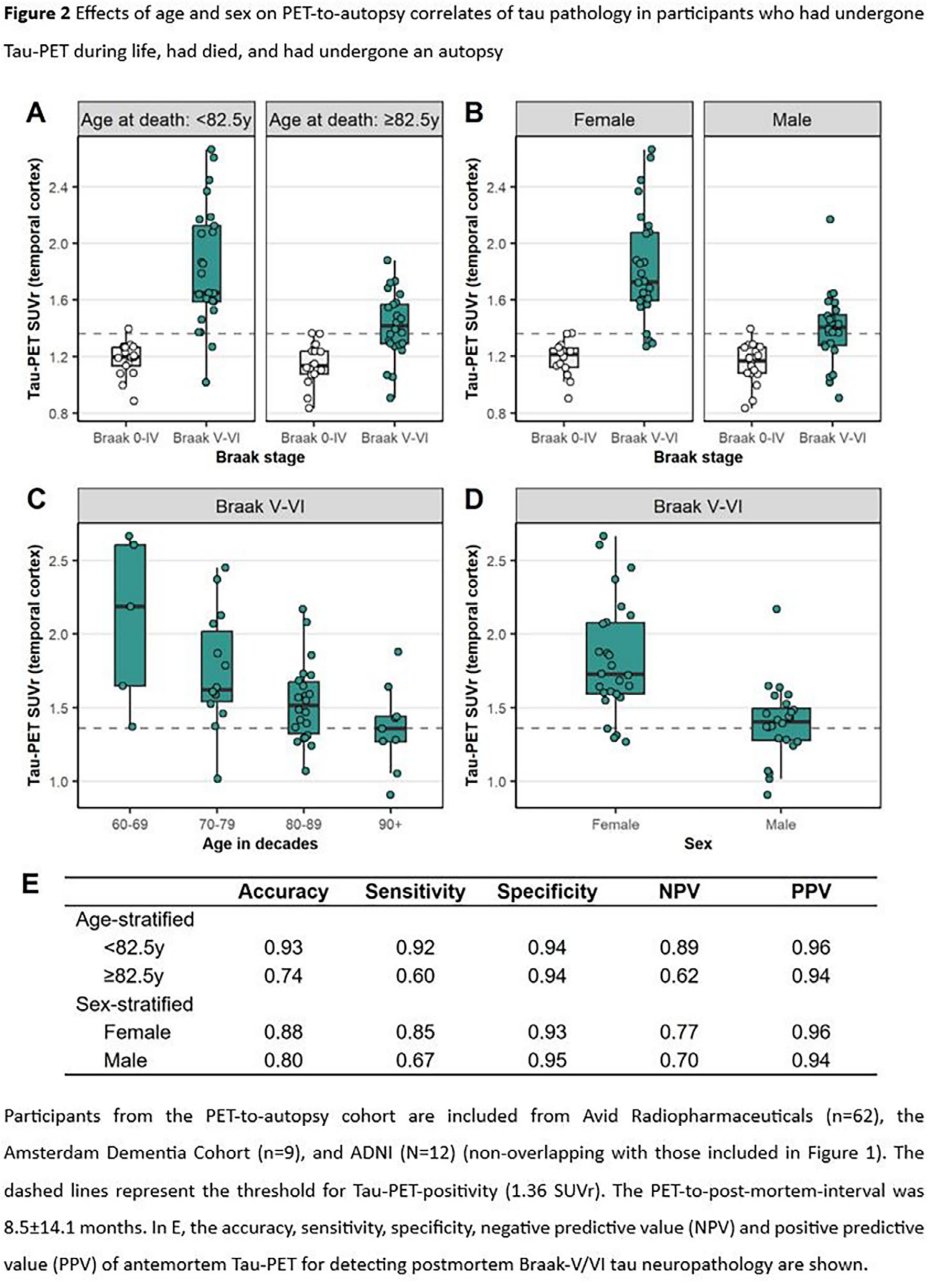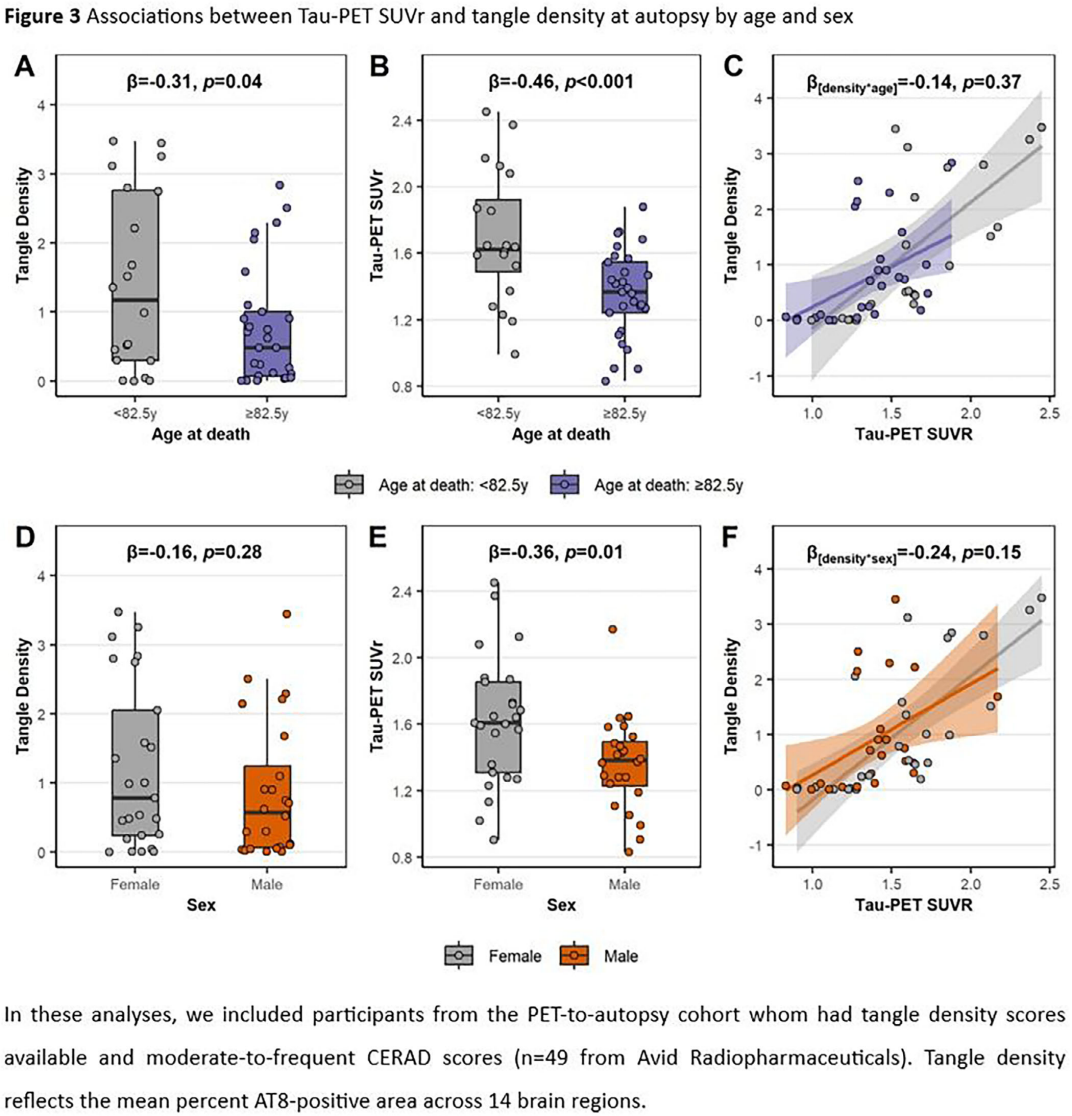# Lower Antemortem [^18^F]flortaucipir PET retention in Males and Older Individuals is Explained by Lower Postmortem Tau Tangle Density

**DOI:** 10.1002/alz70862_110850

**Published:** 2025-12-23

**Authors:** Emma M. Coomans, Ruben Smith, Daria Pawlik, Kevin Oliveira Hauer, Sebastian Palmqvist, Michael Pontecorvo, Sergey Shcherbinin, Vikas Kotari, Geidy E Serrano, Thomas G Beach, Erik Stomrud, Niklas Mattsson‐Carlgren, Annemieke J.M. Rozemuller, Wiesje M. van der Flier, Yolande A.L. Pijnenburg, Elsmarieke van de Giessen, Oskar Hansson, Rik Ossenkoppele

**Affiliations:** ^1^ Department of Neurology, Alzheimer Center Amsterdam, Amsterdam Neuroscience, Vrije Universiteit Amsterdam, Amsterdam Netherlands; ^2^ Amsterdam Neuroscience, Neurodegeneration, Amsterdam Netherlands; ^3^ Clinical Memory Research Unit, Lund University, Malmö, Skåne Sweden; ^4^ Memory Clinic, Skåne University Hospital, Malmö, Skåne Sweden; ^5^ Clinical Memory Research Unit, Lund University, Lund Sweden; ^6^ Clinical Memory Research Unit, Department of Clinical Sciences Malmö, Faculty of Medicine, Lund University, Lund Sweden; ^7^ Eli Lilly and Company, Indianapolis, IN USA; ^8^ Banner Sun Health Research Institute, Sun City, AZ USA; ^9^ Department of Neurology, Skåne University Hospital, Lund Sweden; ^10^ Wallenberg Center for Molecular Medicine, Lund University, Lund Sweden; ^11^ Department of Pathology, Amsterdam Neuroscience, Amsterdam UMC, Amsterdam, Noord‐Holland Netherlands; ^12^ Alzheimer Center Amsterdam, Neurology, Vrije Universiteit Amsterdam, Amsterdam UMC location VUmc, Amsterdam Netherlands; ^13^ Alzheimer Center Amsterdam, Neurology, Vrije Universiteit Amsterdam, Amsterdam UMC location VUmc, Amsterdam, Amsterdam Netherlands; ^14^ Department of Radiology and Nuclear Medicine, Amsterdam UMC, Vrije Universiteit Amsterdam, Amsterdam Neuroscience, Amsterdam Netherlands

## Abstract

**Background:**

Older age and male sex have been associated with lower Tau‐PET uptake in symptomatic Alzheimer’s disease. We investigated the PET‐to‐neuropathological correlates of age‐ and sex‐effects on tau in independent PET (*N* = 680), autopsy (*N* = 947), and PET‐to‐autopsy (*N* = 84) analyses.

**Method:**

Tau‐PET‐analyses included amyloid‐positive participants with MCI or dementia who underwent [^18^F]flortaucipir‐PET. Autopsy‐analyses included MCI or dementia cases with moderate‐to‐frequent CERAD scores and available Braak and tangle density data. PET‐to‐autopsy‐analyses included cases who had undergone Tau‐PET during life, died, and had undergone autopsy, including Braak staging (PET‐to‐post‐mortem‐interval: 8.5±14.1 months). In independent PET‐ and autopsy‐analyses, we investigated age‐ and sex‐effects on Tau‐PET, Braak‐V/VI‐level tau neuropathology and tangle density. In PET‐to‐autopsy analyses, we assessed the correspondence of antemortem Tau‐PET with postmortem Braak‐V/VI neuropathology stratified for age (median‐split at 82.5y) and sex, as well as associations between Tau‐PET and tangle density according to age and sex.

**Result:**

In PET‐analyses (age: 71.9±8.2, 54.3% male), older age and male sex were associated with a lower prevalence of Tau‐PET‐positivity (β=‐0.44, *p*<0.001 and β=‐0.43, *p* = 0.02 respectively) and lower Tau‐PET SUVr (β=‐0.34, *p*<0.001 and β=‐0.10, *p* = 0.007) (Figure‐1A‐D). In autopsy‐analyses (age: 82.7±7.9, 54.7% male), older age and male sex were associated with a lower prevalence of Braak‐V/VI neuropathology (β=‐0.25, *p*<0.001 and β=‐0.40, *p* = 0.005). Among Braak‐V/VI autopsy cases (*n* = 599), older age (β=‐0.38, *p*<0.001), but not male sex (β=‐0.05, *p* = 0.23), was associated with lower tangle density (Figure‐1E‐H). In the PET‐to‐autopsy‐analyses (age: 82.0±8.8, 51.8% male), the specificity of Tau‐PET for detecting Braak‐V/VI tau was high across ages and sexes, but the sensitivity decreased with age and in males (Figure‐2). Older and male participants with moderate‐to‐frequent CERAD showed both lower Tau‐PET and tangle density, and the lack of age/sex‐interactions indicate that the relationship between Tau‐PET and tangle density is consistent across ages and sexes (Figure‐3).

**Conclusion:**

Comprehensive and independent PET, autopsy, and PET‐to‐autopsy analyses demonstrate that the associations between older age and male sex with lower Tau‐PET uptake and prevalence are explained by lower tangle densities at autopsy. Tau‐PET closely reflects postmortem tangle density, which explains the lower sensitivity of Tau‐PET to detect Braak‐V/VI tau at lower densities frequently observed in older and male individuals.